# Proximal radioulnar translocation associated with elbow dislocation and radial neck fracture in child: a case report and review of literature

**DOI:** 10.1007/s00402-013-1820-8

**Published:** 2013-07-25

**Authors:** Hong-Kee Yoon, Gi-Won Seo

**Affiliations:** Department of Orthopaedic Surgery, Soonchunhyang University College of Medicine, 59 Daesagwanro, Yongsangu, Seoul, 140-743 South Korea

**Keywords:** Radioulnar translocation, Radial neck fracture, Elbow dislocation, Mini-open reduction, Child

## Abstract

Proximal radioulnar translocation with radial neck fracture and elbow dislocation is extremely rare. We report a case of a 5-year-old boy who was presented with elbow dislocation, and proximal radioulnar translocation was diagnosed a day after the injury. Mini-open technique was used to reduce the translocation and radial neck fracture. The patient finally regained full range of elbow motion and forearm rotation. This case had clinical importance in that the reverse instability of the elbow was observed compared with the previous reports.

## Introduction

The proximal radioulnar translocation means that both the radius and ulna are dislocated from the humerus. Then, transposed such that the ulna appears opposite to that of the capitellum, and the radial head appears opposite to the trochlea on radiographs [5]. Dislocation of the elbow in children is an uncommon injury and only 3–6 % of all elbow injuries [1]. In addition, proximal radioulnar translocation associated with elbow dislocation is an extremely rare combination of trauma to children. Since MacSween first reported in 1979, only 17 cases have been reported in children [1, 2, 5, 8]. Initial diagnosis is sometimes difficult because the unexpected anatomical relationship is easily overlooked in radiographs [2].

We report an even rarer subtype of this translocation associated with a radial neck fracture and elbow dislocation. This case had clinical importance in that reverse instability was observed compared with the previous reports.

## Case report

**Fig. 1:**
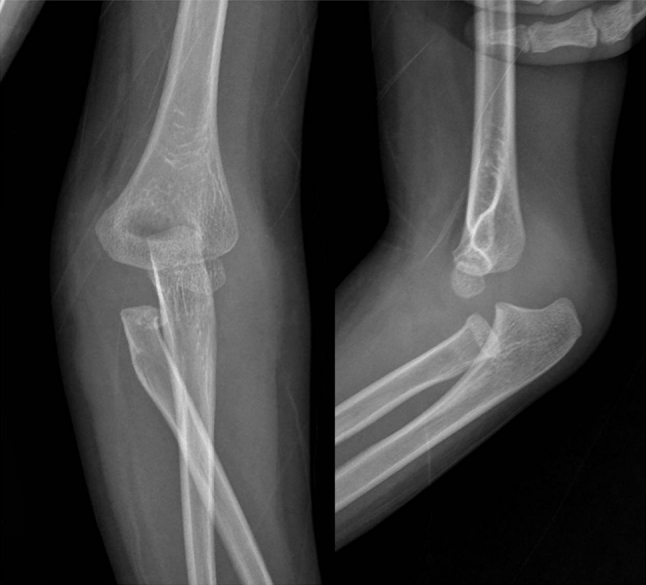
Initial anteroposterior and lateral radiographs show elbow dislocation and translocation of proximal radioulnar joint with radial neck fracture

**Fig. 2:**
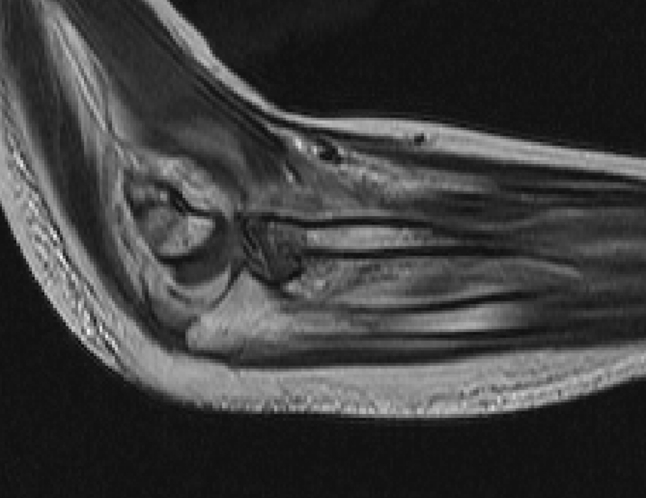
Magnetic resonance image shows the radial head being caught between brachialis tendon and cononoid process

A 5-year-old boy was admitted to our emergency center after a fall from a 2 m jungle gym onto his outstretched left hand. The patient complained of left elbow pain and limitation of motion. Initial examination revealed a painful, swollen left elbow without neurovascular problems. The left elbow was held in 50^o^ flexed position with the forearm in supinated position. The posterior elbow dislocation and radial head fracture were recognized at initial radiographs (Fig. 1). The closed reduction was performed under intravenous sedation and the elbow was immobilized with long arm splint. However, the pain did not subside on the following day and the forearm showed limatation of rotation, especially pronation. The radiographs after reduction were reviewed carefully, and we realized the reversal anatomical relationship of the forearm bone with the humerus. Magnetic resonance image was taken before surgery to evaluate associated soft tissue injury. The radial head was incarcerated between the coronoid process and brachialis insertion (Fig. 2).

**Fig. 3:**
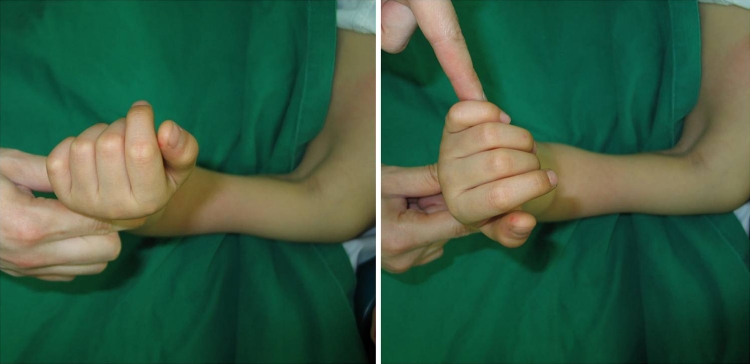
Clinical photographs of forearm rotaion under general anesthesia show limitation of pronation beyond netral position (*left*) comparing with supination (*right*)

**Fig. 4:**
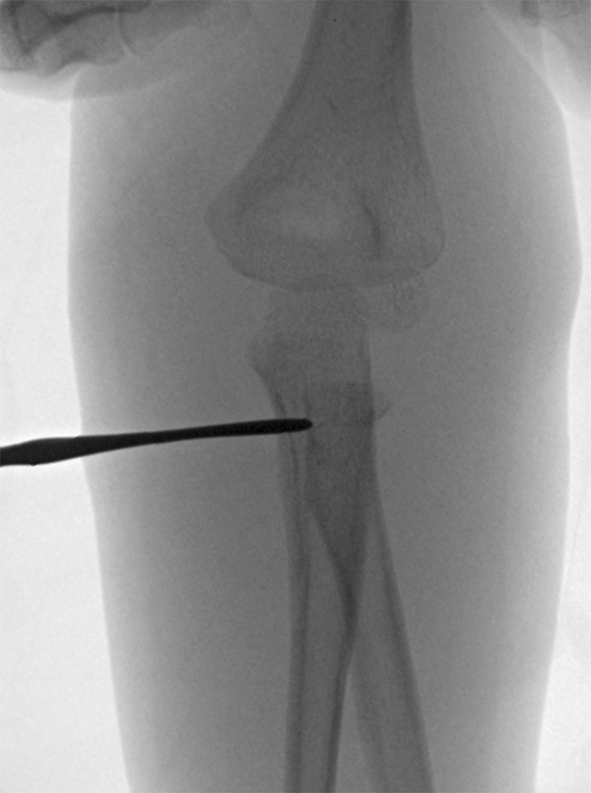
Intraoperative fluoroscopy shows freer pushing the proximal radius laterally

**Fig. 5:**
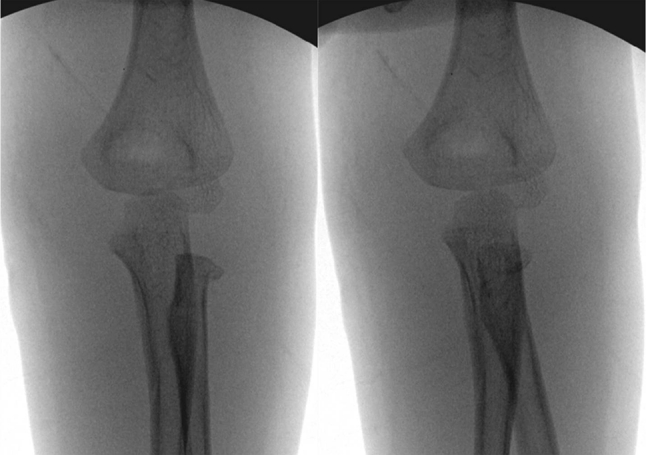
Fluoroscopic images show that the radial head is reduced in pronation (*left*), but subluxated in supination (*right*)

**Fig. 6:**
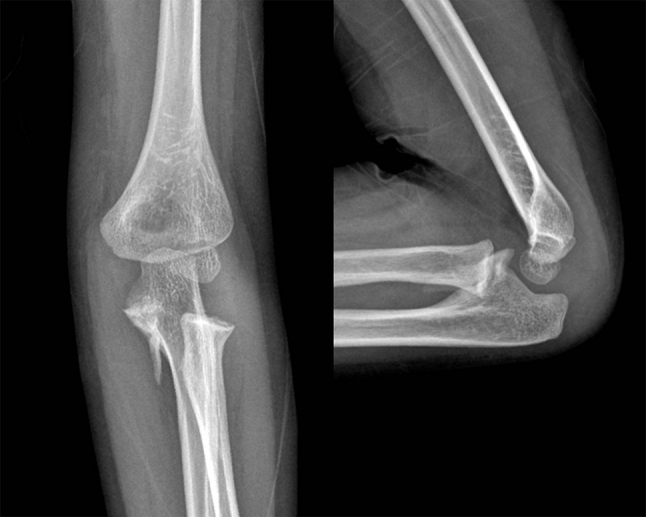
Radiographs at 6 months after the injury show well reduced elbow with calcification at proximal ulna

**Fig. 7:**
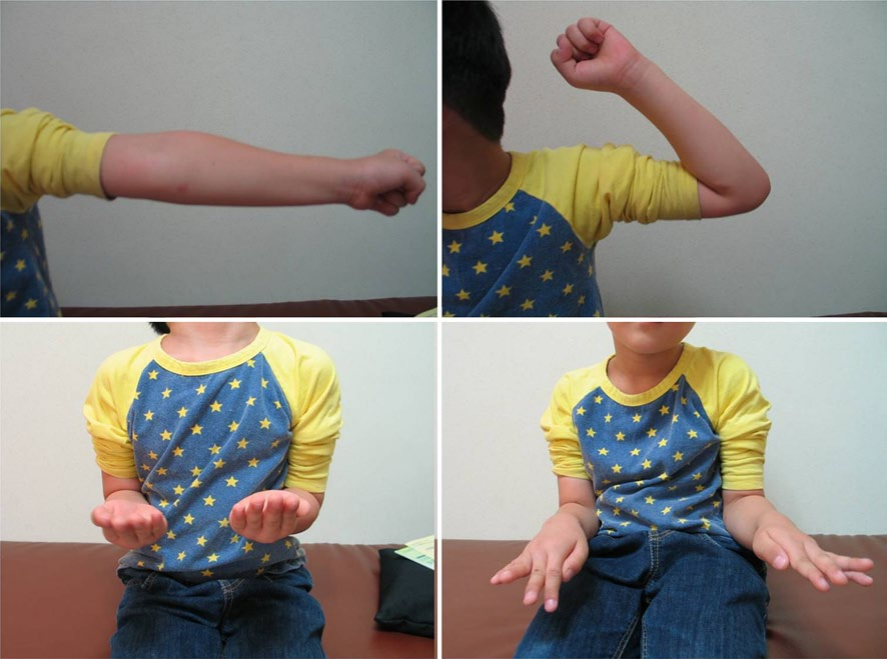
Follow-up clinical photographs 6 months after the injury show full flexion/extension of the elbow and full supination/pronation of the forearm

Closed reduction was planned again. Under general anesthesia, forearm can be extended and supinated, but cannot be pronated beyond the neutral position (Fig. 3). Longitudinal traction and forced supination of the forearm with manual reduction on the radial head failed to achieve reduction. A 1 cm skin incision was made on medial aspect of the proximal ulna. Freer was inserted through the incision, and manual reduction of proximal radius was performed by pushing it laterally (Fig. 4). After reduction, stability of the proximal radius was checked under fluoroscopy. The proximal radius was subluxated in supination of the forearm, but reduced in pronation of the forearm (Fig. 5). After closed reduction for radial neck fracture using freer, the elbow was immobilized at 90^o^ flexion and 45^o^ pronation of the forearm in long arm splint for 4 weeks. At the 2-month follow-up, flexion-extension arc of the elbow was almost recovered; however, forearm rotation is limited yet. At the 6 and 12-month follow-up, the radiographs showed complete healing without pain but heterotopic ossification was observed at anterior aspect of the proximal ulna (Fig. 6). The range of elbow motion and forearm rotation was completely recovered (Fig. 7).

## Discussion

Proximal radioulnar translocation with elbow dislocation, also known as convergent elbow dislocation, is exceedingly rare in children. Ever since MacSween reported the first case, there have been only 17 cases reported [1, 5, 8].

This dislocation can be easily overlooked if the radiographs are not carefully evaluated [4]. In seven cases among the 17 reported cases, there has been a significant delay (from 5 days to 2 months) in recognizing the translocation [1]. There are several reasons for delayed diagnosis. Because the incidence of this injury is very low, a physician does not think about translocation. Clinically, the dislocated elbow is reduced with “clunk” and flexion-extension arc is improved. However, the forearm is locked in almost complete pronation [3]. Radiologically, the lateral view may sometimes give an appearance of a well reduced joint after closed reduction [9]. This finding makes us to overlook the abnormal finding on the anteroposterior view, in which the radius is articulates with the trochlea, while the ulna is articulating with the capitellum [1]. In this case, we also initially missed the translocation. Clinical sign of continuous pain and limited rotation of forearm led us to look at radiographs again, and then translocation could be diagnosed. This follows the pattern in the literature with almost half of the cases being diagnosed late [5]. The key to a correct early diagnosis is extra vigilance and careful inspection of the true anteroposterior and lateral radiographs, as well as clinical suspicion about the possibility of translocation, when clinical signs of restricted forearm rotation and pain continue even after reducing the dislocation.

The mechanism of this injury is a fall onto the hyperpronated outstretched hand, producing an axial load on the proximal aspect of the radius [9]. Combourieu et al.[5] also suggested the mechanism of translocation. The proximal radioulnar translocation is thought to be caused by hyperpronation of the radius with substantial disruption of the ligaments and soft tissues regarding the proximal radioulnar joint, especially the radial collateral ligament and the annular ligament. The radius then spins along the anteriorly through the injured brachialis anterior tendon. MRI of this case shows that the dislocated radial head is incarcerated between the coronoid process and brachialis tendon. The tendinous lesion becomes visible after a few weeks because of heterotopic ossification at the anterior aspect of the ulna [5]. Isbister proposed a different mechanism for translocation [7]. Under axial loading and continuous valgus strain, the ulna passed posterolaterally; whereas the radius crossed over the ulna in the medial aspect of the joint.

Associated injuries are radial head fractures [4, 9], radial neck fracture [8], coronoid process fracture [6] and ulnar nerve palsy [3–5, 7]. Concomitant fractures can complicate the treatment plan, and hence the outcome can be less satisfactory [4, 7, 8]. Growth disturbance of the radial head is a common potential outcome following elbow trauma in children. This finding suggests a disruption of epiphyseal vessel due to the wide displacement of the proximal part of the radius at the time of injury [5]. The clinical finding in this case is different from the previous cases. Limitation of forearm pronation was more prominent than supination before reduction. In addition, subluxation of the proximal radioulnar joint was observed in supination even after reduction. Radial neck fracture was suspected as a reason of different clinical finding and reduction of the radial neck was attempted. However, instability did not improve although neck-shaft angle of the radius was restored.

Successful closed reduction had been reported in only a minority of cases [1]. The reason may be late diagnosis, significant soft tissue interposition preventing reduction, and the presence of associated injuries which necessitate operative intervention. Open reductions through medial or lateral approach were performed in reported cases when the closed reduction failed. We suggest a mini-open technique described in this case report. This method can lessen the additional soft tissue injury to the elbow and chance of heterotopic ossification.

Failure to diagnose this injury early may lead to severe restriction of elbow motion with significant functional impairment [1]. There are two keys to a early diagnosis. First is careful inspection of the true AP radiograph, which shows abnormal articulation between the proximal radioulnar joint and humerus. The second is clinical sign after closed reduction of elbow dislocation, limitation of forearm rotation and elbow motion, and unexplained continuous pain even after reduction.
